# Bright high-repetition-rate source of narrowband extreme-ultraviolet harmonics beyond 22 eV

**DOI:** 10.1038/ncomms8459

**Published:** 2015-06-11

**Authors:** He Wang, Yiming Xu, Stefan Ulonska, Joseph S. Robinson, Predrag Ranitovic, Robert A. Kaindl

**Affiliations:** 1 Materials Sciences Division, E. O. Lawrence Berkeley National Laboratory, MS 2-354, 1 Cyclotron Road, Berkeley, California 94720, USA

## Abstract

Novel table-top sources of extreme-ultraviolet light based on high-harmonic generation yield unique insight into the fundamental properties of molecules, nanomaterials or correlated solids, and enable advanced applications in imaging or metrology. Extending high-harmonic generation to high repetition rates portends great experimental benefits, yet efficient extreme-ultraviolet conversion of correspondingly weak driving pulses is challenging. Here, we demonstrate a highly-efficient source of femtosecond extreme-ultraviolet pulses at 50-kHz repetition rate, utilizing the ultraviolet second-harmonic focused tightly into Kr gas. In this cascaded scheme, a photon flux beyond ≈3 × 10^13^ s^−1^ is generated at 22.3 eV, with 5 × 10^−5^ conversion efficiency that surpasses similar harmonics directly driven by the fundamental by two orders-of-magnitude. The enhancement arises from both wavelength scaling of the atomic dipole and improved spatio-temporal phase matching, confirmed by simulations. Spectral isolation of a single 72-meV-wide harmonic renders this bright, 50-kHz extreme-ultraviolet source a powerful tool for ultrafast photoemission, nanoscale imaging and other applications.

## Abstract (web summary)

Table-top extreme-ultraviolet (XUV) light sources with high repetition rates are sought for fundamental studies, metrology and imaging. Here, Wang *et al*. demonstrate the efficient generation of bright XUV harmonics at 50-kHz repetition rate, with very high photon flux and spectral definition.

## Graphical abstract

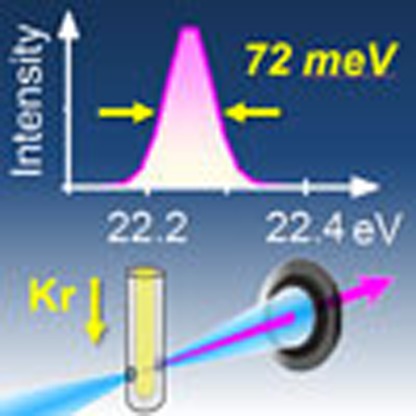

Unique table-top sources of spatially and temporally coherent X-rays are enabled by high-harmonic generation (HHG), which is based on strong-field ionization, acceleration and recombination of electrons in intense laser fields exceeding 10^13^ W cm^−2^ (refs 1, 2, 3, 4). Generally, HHG is driven by energetic, mJ-scale lasers operating at low repetition rates—from a few Hz to several kHz—that allow for loose focusing to maximize phase-matching and thus the conversion efficiency^5^,^6^. Extending table-top extreme-ultraviolet (XUV) sources with ample flux towards rates of ≈50 kHz and beyond is difficult, but can dramatically advance both fundamental investigations of matter and applications in metrology or imaging. For instance, high repetition rates are critical to coincidence and time-of-flight spectroscopy of molecules and solids^7^,^8^, and can also boost photoemission-based imaging and time-resolved studies where electron space-charge effects require spreading the flux over many pulses^9^,^10^,^11^,^12^,^13^,^14^,^15^,^16^,^17^. Material studies in particular require a narrow bandwidth (≲100 meV) to discern the electronic structure, and ≈10^13^ photons s^−1^ (ph s^−1^) HHG source flux before spectral selection for acquisition times comparable to static synchrotron-based photoemission^14^,^18^. Efficient high-repetition-rate HHG, however, is challenging due to the difficulty of phase-matching the conversion in a tight laser focus, necessary to achieve strong-field conditions with μJ-level driving pulses. XUV generation directly from 50 to 100 kHz Ti:sapphire amplifiers so far resulted in ≈10^−8^ conversion efficiency and a flux up to ≈3 × 10^9^ ph s^−1^ per harmonic^19^,^20^,^21^.

Different schemes have been pursued to address this challenge, encompassing a large range of approaches that start from high (50–100 kHz) and extend up to ultra-high (multi-MHz) repetition rates. The latter were enabled by increasing the repetition rate thousand-fold via intra-cavity HHG in enhancement resonators, which boosts the average XUV power despite limited (≈10^−11^–10^−7^) conversion efficiencies^22^,^23^. An optimized setup delivers 10^12^–10^13^ ph s^−1^ harmonic flux around 50 MHz (ref. 24). Although ideal for XUV frequency-comb metrology, these oscillator-based schemes are unsuited to ultrafast studies requiring strong excitation pulses and continuous operation is limited by hydrocarbon contamination at kW intra-cavity powers. Alternatively, high-repetition-rate HHG directly with intense driving pulses is enabled by Yb-based solid-state or fibre amplifiers that withstand high average powers^25^,^26^,^27^. At 100 kHz repetition rate, 10^12^ ph s^−1^ were generated, which corresponds to ≈5 × 10^−7^ efficiency^26^. Scaling up to 0.6 MHz was achieved by combining the output of multiple fibre amplifiers to 163 W power, yielding more than 10^13^ ph s^−1^ flux with up to 2 × 10^−6^ efficiency^28^. Recently, the absorption limit of infrared-driven HHG was reached using 8 fs pulses and gas pressures up to several bar, yielding broad harmonics with 8 × 10^−6^ efficiency and ≈2 × 10^12^ ph s^−1^ flux at 150 kHz (ref. 29). In this context, further enhancement of high-repetition-rate HHG motivates the investigation of methods to boost the conversion process itself. Below-threshold harmonics represent one possibility, where phase matching near atomic resonances enables efficient, yet spectrally broad and structured emission^30^. A second, highly interesting route arises from strong wavelength scaling of the HHG atomic dipole, evidenced at low repetition rates by increased XUV flux resulting from mJ-scale, visible and ultraviolet pulses loosely focused for optimal phase matching^31^,^32^,^33^,^34^. Efficient HHG with short-wavelength sources, however, has so far not been demonstrated under high-repetition-rate conditions, and HHG sources are not typically optimized to generate spectrally narrow harmonics.

Here, we explore ultraviolet-driven HHG in the tight-focusing regime and establish a highly-efficient source of narrowband XUV pulses at 50-kHz repetition rate. A bright harmonic flux of ≈3 × 10^13^ ph s^−1^ is generated at 22.3 eV in this cascaded approach, where efficient frequency doubling is followed by HHG in Kr gas. We establish an XUV conversion efficiency of up to ≈5 × 10^−5^, which exceeds by two orders-of-magnitude that of similar harmonics driven directly by the near-infrared laser amplifier. This strong boost surpasses the dipole wavelength scaling and, as confirmed by numerical simulations, evidences enhanced spatio-temporal phase matching for ultraviolet-driven harmonics in the sharply focused beam. The spectral structure enables direct isolation of a single, 72-meV wide harmonic—yielding a compact and bright, high-repetition-rate XUV source for a new class of ultrafast XUV studies.

## Results

### High-repetition-rate XUV generation

**Figure 1:**
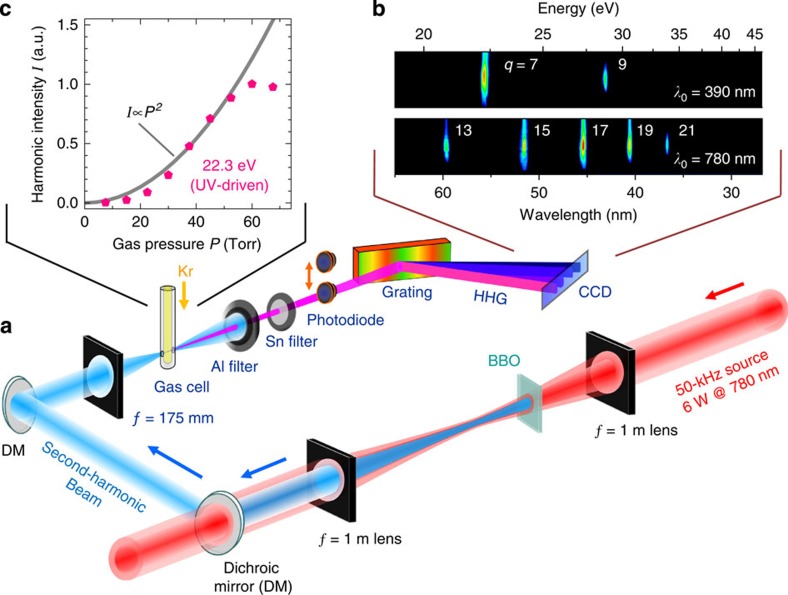
Efficient high-repetition-rate source of extreme-ultraviolet (XUV) pulses. (**a**) Scheme for two-stage high-harmonic generation, starting from 120 μJ near-infrared pulses at 50-kHz repetition rate, which in the first step are frequency doubled to 390 nm wavelength in BBO. These ultraviolet (UV) pulses are subsequently focused sharply onto a thin column of Krypton gas to initiate high-harmonic generation. The resulting XUV light is filtered with thin metal foils, followed by photon flux and spectral characterization with a calibrated XUV photodiode and grating spectrometer. (**b**) Intensity-normalized CCD images of the spectrally dispersed *q*-th XUV harmonics, generated by either the UV pulses or the near-infrared fundamental at their respective driving wavelength *λ*_0_. (**c**) Scaling of XUV intensity with Kr gas pressure, for the brightest UV-driven harmonic at 22.3 eV.

Our scheme is illustrated in Fig. 1a. Near-infrared pulses of 120 μJ energy and 50-fs duration are generated by a cryogenically cooled, high-repetition-rate (50 kHz) Ti:sapphire regenerative amplifier, and focused onto a 0.5-mm-thick β-Barium borate (BBO) crystal for frequency doubling with ≈40% efficiency. Here, loose focusing with a *f*=1 m focal length lens avoids nonlinear spectral broadening and ionization in the air. This results in ultraviolet pulses centred around 390 nm wavelength with 48 μJ pulse energy, which are separated from the fundamental via two dichroic multilayer mirrors. To initiate the HHG process, the femtosecond ultraviolet pulses are recollimated and subsequently focused sharply via a *f*=175 mm lens onto a cylindrical gas cell, consisting of an end-sealed glass capillary housed in a vacuum chamber. The capillary is supplied with Kr gas and positioned near the focus of the second-harmonic laser beam, while suppressing the production of off-axis beams arising from long-trajectory electron dynamics^35^.

Under these conditions we observe the emission of strong XUV harmonics. Their spectral content is characterized by a spectrometer after blocking the diverging optical beam with thin aluminum filters (see Methods). Figure 1b (top image) shows the resulting charge-coupled device (CCD) readout for a gas pressure of 60 Torr. Bright peaks are detected around 22.3 and 28.6 eV, respectively, corresponding to the 7th and 9th harmonics of the ultraviolet driving pulses. For direct comparison, we also recorded the XUV emission generated by directly focusing the ≈780 nm fundamental pulses onto the Kr gas to a similar peak intensity. Multiple harmonics are observed, as shown in the corresponding normalized CCD data in Fig. 1b (bottom image). However, the emission was found to be significantly weaker compared with the ultraviolet-driven harmonics.

Figure 1c shows the dependence of the XUV intensity of the most intense harmonic on the backing pressure of the Kr gas target, indicating a rapid nonlinear increase up to about 60 Torr followed by saturation at higher pressures. The strong XUV emission arises from phase-matched harmonic generation, because of in-phase coherent addition of the XUV fields emitted from the Kr atoms. At higher pressures, plasma defocusing limits the overall yield^20^,^36^,^37^. We have also generated harmonics in Ar gas for comparison, resulting however in ≈5 times lower yield. For optimal phase-matching in Kr, the vertical intensity contour on the CCD image (Fig. 1b) perpendicular to the dispersion direction indicates an approximately Gaussian beam profile. From its extent, we obtain a beam divergence of 6 mrad (full-width at half-maximum, FWHM), which facilitates extended beam propagation for refocusing or additional optical manipulations.

### Spectral structure and harmonic flux

**Figure 2:**
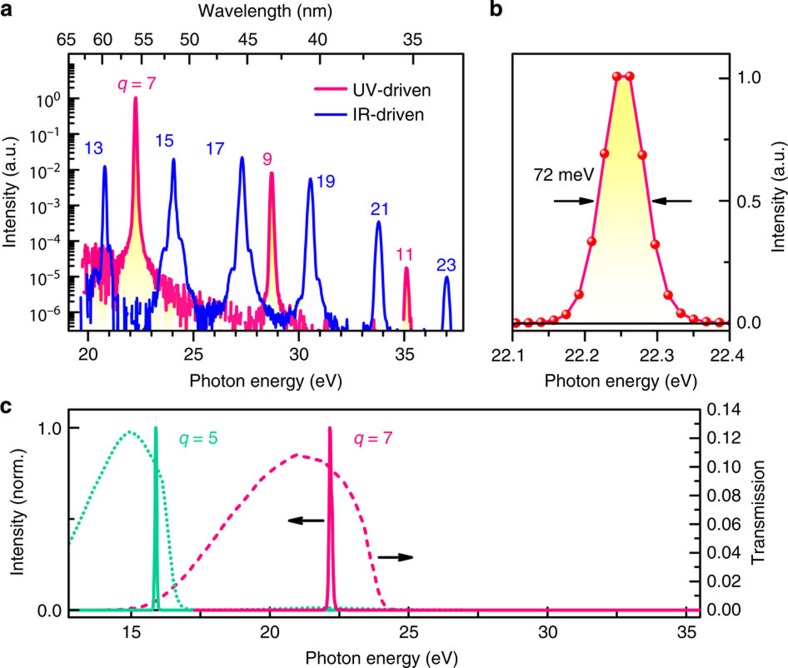
XUV spectra and isolation of a single harmonic. (**a**) Spectra of the XUV harmonics (vertical CCD lineouts) driven either by the 48-μJ ultraviolet (UV) pulses or by the 120-μJ pulses from the near-infrared (IR) laser fundamental. Spectra are measured for a Kr gas pressure of 60 Torr, and are shown for the same integration time of 4 s and with CCD background noise subtracted. For the ultraviolet-driven harmonics, the spectrum was corrected for the effect of one additional Al filter, inserted to avoid CCD saturation. (**b**) Emission profile of the 7th harmonic at 22.25 eV with corresponding line width (FWHM). (**c**) Isolated single harmonics (solid lines) after absorptive spectral filtering with thin metal foils. The 7th-harmonic is isolated via combined Sn and Al filters of 300-nm thickness each, whereas the 5th-harmonic is selected by an In foil in combination with Brewster reflection from two Si plates^38^ to suppress the residual laser beam. For comparison, the theoretical transmission is shown for Sn (dashed line) and In (dotted line) of 300-nm thickness^49^.

The experiments reveal a striking enhancement of the XUV emission generated by the 390-nm field as compared with that obtained from the near-infrared fundamental. Figure 2a compares the measured XUV spectra for the same integration time, corrected for an additional Al filter used to avoid CCD saturation. The spectra encompass a series of odd harmonics that span photon energies up to ≈37 eV with strongly varying peak intensities, where the 7th harmonic of the ultraviolet field constitutes by the far strongest XUV emission. The latter, ultraviolet-driven peak at ≈22.3 eV surpasses that of the spectrally similar 13th and 15th harmonics of the near-infrared fundamental by 50–80 times. This strong enhancement is particularly striking, as it occurs despite the necessarily lower energy of the ultraviolet pulses derived from frequency doubling. Importantly, this harmonic exhibits a linewidth as narrow as 72 meV FWHM near optimum pressure, as shown in Fig. 2b. This yields a critical advantage for applications such as photoelectron spectroscopy or zone-plate imaging.

The ultraviolet-driven XUV generation entails a large energy separation of ≈6.4 eV between the individual odd harmonics. We can exploit this distinctive feature to isolate a single harmonic from the comb, taking advantage of the atomic and plasma absorption edges of thin metal foils. To select harmonics around 22 eV, the beam is passed through 300-nm-thick Sn, whose transmission is indicated by the dashed line in Fig. 2c and which strongly attenuates the adjacent 5th and 9th harmonics. Together with the Al foil used to block the residual visible beam, for a combined XUV transmission of ≈1%, we obtain the spectrum in Fig. 2c (magenta line). In this scheme, the 5th and 3rd harmonics at lower energies are also suppressed via the Al foil that blocks the ultraviolet driving field. By attenuating the laser beam instead via two Brewster's-angle reflections from silicon^38^, the lower-order harmonics are transmitted. The 5th harmonic at 15.9 eV is then spectrally selected using a 300-nm-thick In foil, as demonstrated in Fig. 2c (green line), with a flux and bandwidth comparable to the isolated *q*=7 harmonic. Thus, high-contrast isolation of individual ultraviolet-driven harmonics is achieved at either 15.9 or 22.3 eV, resulting in a compact source of narrowband and single-harmonic XUV pulses without the need for a complex monochromator.

To obtain the absolute XUV photon flux for each harmonic, the total source power was determined with a calibrated X-ray photodiode, taking into account the filter transmission, and then split according to the harmonic ratios (see Methods). Table 1 shows the photon flux of the individual harmonics and resulting energy conversion efficiencies. Up to 3.3 × 10^13^ ph s^−1^ are generated in the 7th harmonic of the ultraviolet-driving pulses, corresponding to 117 μW source power emitted from the Kr gas at 22.3 eV. The corresponding HHG efficiency (5 × 10^−5^) is ≈140–400 times higher than for the spectrally closeby 15th and 13th harmonics of the fundamental. The values must be compared at similar photon energies due to the wavelength-dependent opacity of the gas^39^. By contrast, when the influence of phase-matching can be neglected, a scaling ∝*λ*_0_^−4.7±1^ with visible driver wavelength was previously found^40^, which contributes a ≈12 to 52-fold increase upon frequency doubling of *λ*_0_. This comparison underscores the significant phase-matching advantage of the ultraviolet-driven HHG under our experimental conditions.

### Phase-matching simulations of ultraviolet-driven HHG

In the following, we present numerical HHG simulations to clarify the phase-matching boost of ultraviolet-driven HHG with our experimental parameters. The generated number of XUV photons in the *q*-th harmonic can be expressed as^37^ *N*_*q*_∝*β*_S_(*q*,*ω*_0_) × *ξ*_*q*_. Here, *β*_S_ is the single-atom efficiency due to electronic wavepacket dynamics, which approximately follows *β*_S_*∝ω*_0_^5^ around the cutoff due to wavepacket quantum diffusion and energy scaling^41^. In turn, *ξ*_*q*_ is the enhancement factor arising from the spatio-temporal folding of XUV emission and phase matching^5^,^37^. In the simulation, the XUV flux generated from each temporal slice of the driving field results from the coherent superposition of all harmonic emissions, integrated across the interaction volume^5^. For this, we take into account the XUV generation at each location *z*, reabsorption by the Kr gas, ground state depletion and the wavevector mismatch between the high-harmonic and driving fields


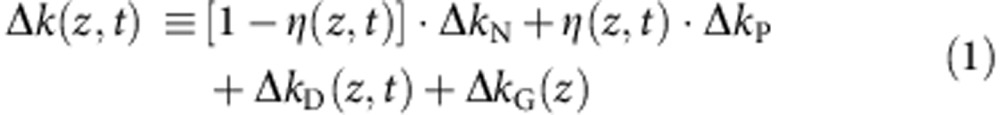


The first two terms above describe the mismatch due to dispersion at a given pressure, where Δ*k*_N_ corresponds to the charge-neutral gas and Δ*k*_P_ to a fully ionized plasma, accordingly scaled by the ionization level *η*. Moreover, Δ*k*_D_ results from the atomic dipole phase accumulated by the accelerated electron wavepacket, and Δ*k*_G_ from the geometric Gouy phase of the focused beams.

For loose focusing attainable with mJ pulses, the spatial dependence and Gouy-phase contribution are negligible. Phase matching is then achieved at suitable pressures by dynamically balancing to Δ*k*≈0 via the ionization level, given that the sign of the plasma contribution (Δ*k*_P_>0) is opposite to Δ*k*_N_ of neutral atoms. Such conditions are fulfilled only during part of the driving pulse, as the ionization level rises quickly with time^3^. Instead, under tight focusing where the Rayleigh length *z*_R_ is comparable to the gas cell thickness, the conversion efficiency is reduced due to the added spatial dependence of Δ*k*. The Gouy term now becomes significant and as its sign equals the plasma contribution, phase-matching occurs at a lower ionized fraction and thus reduced intensity. Although increased pressures can partly compensate this reduction, the tolerable gas density is limited by plasma defocusing and pumping capabilities^36^,^37^,^42^.

**Figure 3:**
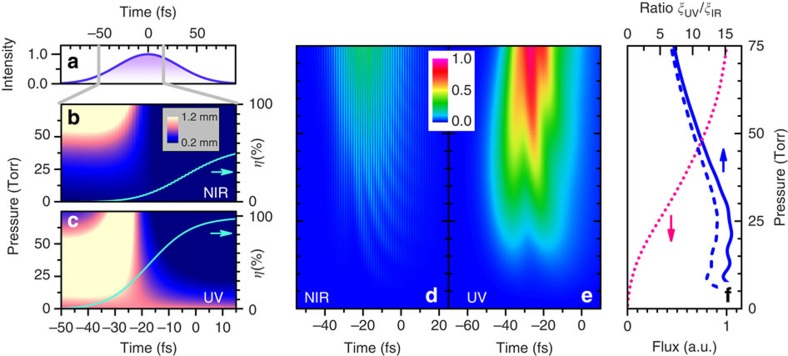
HHG phase matching simulations in the tight focus geometry. The calculations are for a 75-fs FWHM driving pulse with 1.8·10^14^ W cm^−2^ peak intensity, Rayleigh length of *z*_R_=1 mm and with the gas volume centred at *z*=*z*_R_/2 after the focus. (**a**) Driving pulse intensity. (**b**,**c**) Coherence length *π*/|Δ*k*|, mapped as function of time and Kr gas pressure, comparing the phase-matching at similar XUV photon energies of the near-infrared (NIR)-driven 15th and ultraviolet (UV)-driven 7th harmonic. Plots are shown at 200 μm before the gas volume exit, comparable to the XUV absorption length at 50 Torr. The Kr ionization level is shown for comparison (blue line) as calculated with the YI model^43^. (**d**,**e**) Resulting XUV photon flux enhancement, obtained by spatially integrating the HHG emission across the interaction volume (see Supplementary Methods) for the two cases, with the single-atom efficiency *β*_S_ omitted. The flux enhancement is mapped as a function of gas pressure and emission time within the driving pulse. (**f**) Pressure dependence of the UV-driven XUV emission (dotted line), obtained by integrating the data in **e** across the pulse. The ratio of the time-integrated phase-matching enhancements *ξ*_UV_/*ξ*_IR_ is shown for the above model (dashed line), and for better comparison with experiment using a 50-fs NIR driving pulse (solid line).

When ultraviolet-driving pulses are employed, the above spatio-temporal effects of phase-matching on the XUV emission—represented by the enhancement *ξ*_*q*_—are substantially improved. We have calculated phase-matched HHG in Kr gas with the above model under our conditions, for pulses with either 390 or 780 nm centre wavelength and otherwise identical 1.8 × 10^14^ W cm^−2^ peak intensity and 75-fs duration (for details see Supplementary Methods). To mimic the experiment, we further consider a Rayleigh length of *z*_R_=1 mm and a 1-mm-thick gas volume. The driving pulse profile is indicated in Fig. 3a. Figure 3b,c maps the coherence length *L*_coh_=*π*/|Δ*k|* as a function of gas pressure and time. The Kr ionization levels are shown for comparison (lines in Fig. 3b,c) and ramp up quickly towards the pulse centre. They are calculated with the Yudin-Ivanov model^43^ to encompass both tunnelling and multi-photon ionization, whose relative influence was illustrated in previous studies of HHG phase-matching and wavelength scaling under loose focusing^44^. Several aspects contribute to the enhanced phase-matching in the ultraviolet field. First, due to 1/*ω*_0_^2^ scaling of the plasma mismatch, higher ionization levels are tolerated in the ultraviolet as evident from a comparison of Fig. 3b,c. The ionization rates are accordingly higher, and our calculations indicate a ≈4.2 times enhancement over infrared-driven HHG. Second, the spatial phase matching conditions are also improved, as the Gouy phase is proportional to the harmonic order. As a result, the absolute value and spatial dependence of Δ*k*_G_ are reduced by half when driven by the second-harmonic. For these ultraviolet-driven enhancements, we note that plasma scaling equally applies to loose focusing, whereas the Gouy phase reduction only matters for tight focusing entailed by limited pulse energies of high-repetition-rate lasers.

To obtain the total XUV flux, we integrate the harmonic emission across the gas volume, taking into account the gas density, XUV emission and re-absorption, and the spatio-temporal dynamics of the ionization level and phase mismatch. Figure 3d,e compares the resulting XUV flux enhancement beyond the single-atom efficiency *β*_S_, for the two incident wavelengths and mapped out for different pressures and temporal slices within the driving field. This illustrates the time window of XUV emission and demonstrates the clear phase-matching advantage of ultraviolet-driven HHG. Figure 3f shows the pressure dependence of the integrated XUV flux generated by the ultraviolet field (dotted line). The ratio of the total, time-integrated phase-matching enhancements *ξ*_*q*_(UV)/*ξ*_*q*_(IR) is shown in Fig. 3f (dashed line). Further improvement is found when considering the shorter ≈50-fs infrared pulses in the experiment (solid line), resulting in a 10–16 times ultraviolet-driven boost of phase-matched XUV generation below 60 Torr. Combined with the *≈ω*_0_^5^ scaling of the single-atom efficiency that contributes an additional factor of 32, this ultraviolet-driven boost of phase-matched XUV generation explains the two orders-of-magnitude increase in HHG efficiency observed in our experiments.

## Discussion

The efficient HHG conversion demonstrated here at 50 kHz results in a unique XUV source with major experimental benefits. Beyond enhancing signal averaging and statistics in numerous applications, high repetition rates are particularly valuable to advancing photoemission electron microscopy^17^ or angle-resolved photoelectron spectroscopy (ARPES)^11^,^13^, where space-charge Coulomb interactions between the emitted electrons limit acceptable pulse energies. Moreover, in ARPES the accessible momentum space increases rapidly with photon energy, and XUV photons beyond 20 eV render the full Brillouin zone of most materials easily accessible.

After high-contrast spectral isolation of the 22.3 eV harmonic with the combined Al-Sn filter, our femtosecond source delivers ≈3 × 10^11^ ph s^−1^ at the sample. This flux is comparable to continously emitting Helium lamps and even approaches that of monochromatized synchrotron beamlines employed for static ARPES^14^,^18^. As space-charge broadening typically restricts each pulse to ≈10^6^ photons, the 50-kHz repetition rate at this flux provides ideal conditions for photoemission studies^14^,^45^. We note that by utilizing only the Al foil, even higher flux beyond ≈3 × 10^12^ ph s^−1^ is obtained at the expense of isolation contrast. The addition of the Sn filter, however, also provides a compact way to separate the gas-based XUV source and attached optics chambers from a subsequent ultrahigh-vacuum environment.

Spectral isolation and narrowing of high-order harmonics is often achieved using XUV monochromators, which add significant complexity and require non-traditional layouts for time-resolved applications to minimize grating-induced pulse broadening^46^,^47^. In contrast, the ultraviolet-driven HHG source reported here demonstrates the direct generation of narrow harmonics, whose large energy spacing allows for straightforward selection with absorptive metal filters. The measured 72-meV harmonic width corresponds to a ≈0.3% fractional energy bandwidth directly from the source. This intrinsic resolution is well adapted to discerning the electronic structure of atoms, molecules and complex materials via photoemission studies.

Hence, XUV conversion with high efficiency is achieved at 50-kHz repetition rate, resulting from both wavelength scaling of the atomic dipole and enhanced phase-matching conditions in a ultraviolet field under tight focusing conditions. Although based on Ti:sapphire-amplified pulses, this cascaded scheme is generally applicable and attractive for enhancing HHG also with other high-repetition-rate sources. In particular, combining ultraviolet-driven HHG with novel high-power solid-state^48^ or Yb-fibre amplifiers^25^,^26^,^28^ may help scale XUV generation to even higher flux and repetition rates. Finally, additional narrowing of the harmonics would propel scientific studies of low-energy correlations in solids, which motivates further HHG studies with longer or spectrally shaped ultraviolet fields. We expect that the compact 50-kHz source of bright and narrowband XUV harmonics, established here, will boost applications in photoemission and nanoscale imaging, paving the way for novel insights into complex matter.

## Methods

### Femtosecond ultraviolet-driving pulses

The initial stage of the setup is a high average power cryo-cooled Ti:sapphire regenerative amplifier (KMLabs Wyvern 500) that provides near-infrared pulses of 50 fs duration at 50-kHz repetition rate, with a high beam quality (*M*^*2*^=1.3 × diffraction limited). After splitting off half of the output for photo-excitation in time-resolved applications, the remaining pulses with ≈120 μJ energy were focused onto a 0.5-mm-thick BBO crystal with a *f*=1 m lens. The crystal is cut for phase-matched second-harmonic generation (*θ*=29.2°) and positioned 380 mm before the focus, corresponding to a peak intensity of ≈100 GW cm^−2^. We estimate a ultraviolet pulse duration of ≈73 fs by taking into account nonlinear propagation in BBO (using the code SNLO) and pulse dispersion in the focusing optics. Pulses with 3 nm bandwidth are generated around 390 nm wavelength with ≈39.5% conversion efficiency.

### Generation and spectral characterization of XUV harmonics

For HHG, Kr gas is introduced into the end-sealed glass capillary positioned within a vacuum chamber. Here, a 1-mm inner diameter and 100-μm wall thickness of the capillary was chosen to accomodate the *z*_R_≈1 mm Rayleigh length of the driving beam. Before use, beam entry and exit holes are laser-drilled *in situ* at ambient pressure, perpendicular to the capillary side walls via the femtosecond ultraviolet laser pulses. Under normal operation, a 750 l s^−1^ turbopump maintains the pressure in the HHG chamber below 1 mTorr. The capillary backing pressure is monitored with a Si diaphragm gauge.

For XUV generation, the ultraviolet pulses are re-collimated with a *f*=1-m fused silica lens and then focused onto the gas cell with a *f*=175 mm lens, resulting in a 18-μm FWHM beam diameter and *I*_0_≈1.8 × 10^14^ W cm^−2^ peak intensity. Using the fundamental for harmonic generation, these values are 33 μm and *I*_0_≈1.9 × 10^14^ W cm^−2^. After the generation chamber, the intense driving beam is blocked and the XUV harmonics are spectrally selected via thin metal filters, mounted on two gate valves to enable their insertion. The XUV spectra are recorded with an evacuated spectrometer (McPherson 234/302) equipped with a back-illuminated X-ray CCD and a 2,400 l mm^−1^ Pt-coated aberration-corrected concave grating. The CCD covers the 25–75 nm range (≈49.6–16.5 eV) at 50 nm central wavelength, and a 5-μm wide entrance slit is used for ≈0.1 nm spectral resolution.

### XUV photon flux

The absolute photon flux for each harmonic is quantitatively determined by first recording the spectrally integrated XUV flux with a Si photodiode calibrated in this range (IRD AXUV100G), and then splitting this total flux according to the relative ratios of the harmonics. Residual leakage at the drive wavelength is removed by subtracting the background measured without the noble gas. The total charge collected by the photodiode is the sum of contributions from each *q*-th odd harmonic, that is,


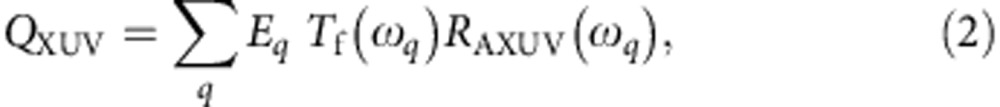


where *E*_*q*_ is the pulse energy within the *q*-th harmonic as emitted at the source, *ω*_*q*_ is the harmonic frequency, *T*_f_ (*ω*) is the Al filter transmission and *R*_AXUV_(*ω*) the calibrated photodiode responsitivity. The Al filter was calibrated *in situ* by measuring the transmission of near-infrared-driven harmonic peaks on the X-ray CCD (Supplementary Fig. 1), with the foil inserted with a gate valve. The absolute values of *E*_*q*_ are then obtained from *Q*_XUV_ using the relative harmonic ratios. These ratios are determined from the CCD spectrum of the XUV harmonics, corrected for filter transmission and grating efficiency, that is, from the count rate


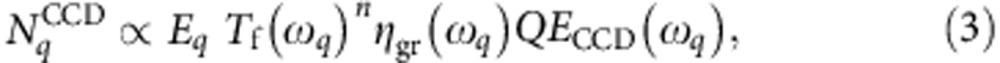


where *η*_gr_(*ω*) is the grating diffraction efficiency provided by the manufacturer and *QE*_CCD_(*ω*) is the CCD quantum efficiency. To avoid saturation of the CCD camera, the XUV flux was suppressed with either *n*=1 or 2 filters for the infrared- and ultraviolet-driven harmonics, respectively. The above procedure thus provides the pulse energy and the photon flux *E*_*q*_/*ħω*_*q*_ for each harmonic.

## Additional information

**How to cite this article:** Wang, H. *et al*. Bright high-repetition-rate source of narrowband extreme-ultraviolet harmonics beyond 22 eV. *Nat. Commun.* 6:7459 doi: 10.1038/ncomms8459 (2015).

## Supplementary Material

Supplementary Information Supplementary Figure 1, Supplementary Methods and Supplementary References.

## Figures and Tables

**Table 1:** Source photon flux and energy conversion efficiency per harmonic.

**Harmonic order**	**Photon energy (eV)**	**Photon flux (ph s^−1^)**	**Conversion efficiency**
*Near-infrared driving pulse (*λ*=780 nm)*			
13th	20.8	2.2 × 10^11^	1.2 × 10^−7^
15th	24	5.3 × 10^11^	3 × 10^−7^
17th	27.3	1.6 × 10^12^	1.2 × 10^−6^
19th	30.5	1.5 × 10^12^	1.2 × 10^−6^
21st	33.7	1.5 × 10^11^	1.3 × 10^−7^
23rd	37	1.9 × 10^9^	2 × 10^−9^
			
*Ultraviolet driving pulse (λ*=*390 nm)*			
7th	22.3	3.3 × 10^13^	5 × 10^−5^
9th	28.7	1.7 × 10^12^	3 × 10^−6^
11th	35	8.3 × 10^9^	2 × 10^−8^
