# Supplementary material for: Bright high-repetition-rate source of narrowband extreme-ultraviolet harmonics beyond 22 eV
Source: Nat Commun. 2015 Jun 11;6:7459. doi: 10.1038/ncomms8459 (PMC4490359; doi:10.1038/ncomms8459)
Supplement: Supplementary Information — Supplementary Figure 1, Supplementary Methods and Supplementary References. [file ncomms8459-s1.pdf]

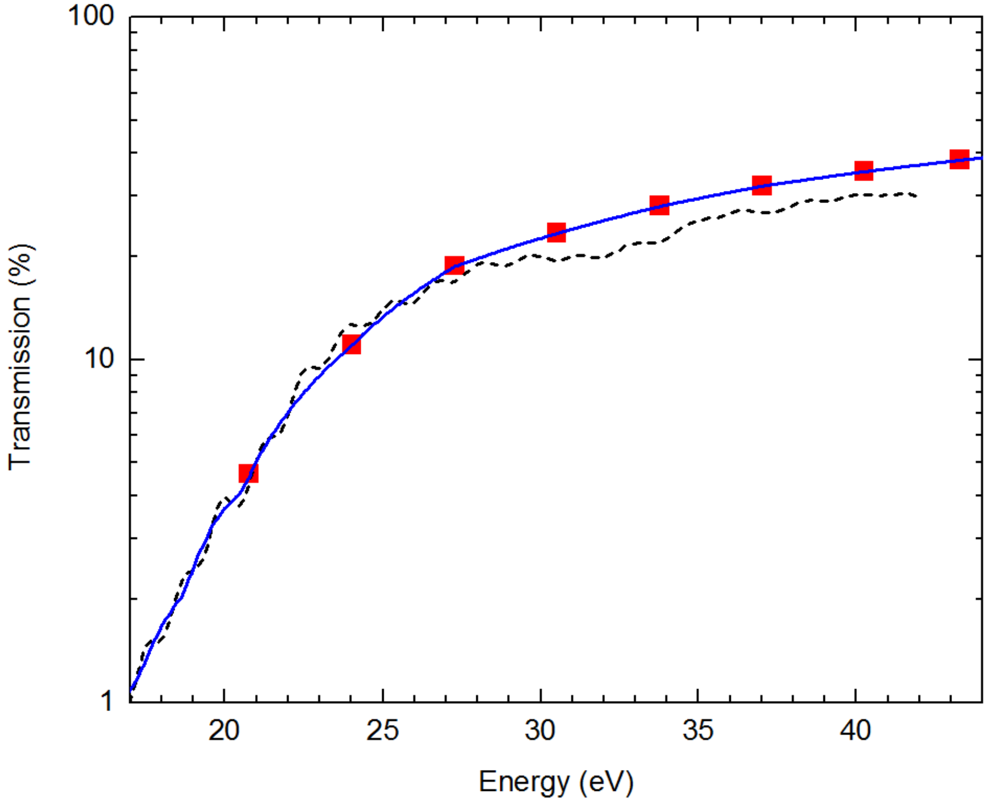

Supplementary Figure 1 | Aluminum Filter Transmission. Transmission of a 300-nm thick, free-standing Al foil used in the experiments (squares). The values are determined from the attenuation of near-IR driven harmonic peaks measured with the spectrometer, after mounting the filter on a gate valve to allow for insertion and removal in the XUV beam. Solid line: interpolated curve as used in the data evaluation described in the main paper. Dashed line: Simulated XUV transmission of a 300-nm thick Al filter including an oxide layer of 45 Å thickness on each side, for comparison. The transmission is calculated by taking into account multiple reflections within a transfer-matrix formalism, based on experimentally-determined optical response functions of Al and Al_2O_3 in the XUV.[1–3]

Supplementary Methods

Model of Phase-matched High Harmonic Generation

The generation of XUV harmonics was simulated by taking into account the time- and spatially-dependent ionization and phase mismatch during the interaction of the intense driving field with a gas of Krypton atoms. The numerical calculations are based on a one-dimensional model of wave-propagation and on-axis strong-field harmonic emission. The corresponding plane-wave electric field for a given frequency ω and wavevector k is expressed as $E(z,t) = \sqrt{2I(z,t)/c_0\varepsilon_0} \times \mathrm{Re}\{\exp[i(\omega t - kz)]\}$. Here, c_0 is the light speed and ε_0 the vacuum permittivity, with all equations in SI units. The intensity of the UV (or near-infrared) driving field is described by the Gaussian envelope

$$I(z,t) = \frac{I_0}{1+(z/z_\mathrm{R})^2}\exp[-4\ln 2\left(\frac{t}{\tau}\right)^2], \tag{1}$$

where I_0 is the peak intensity, τ is the full-width at half-maximum (FWHM) pulse duration, and z_R the Rayleigh length.

Wavevector mismatch. To take into account the phase mismatch during HHG, we define the wavevector difference

$$\Delta k \equiv k(q\omega_0) - qk(\omega_0) \tag{2}$$

between the q-th harmonic at frequency $q\omega_0$ and the fundamental at ω_0. This mismatch is the sum of several contributions:[4]

$$\Delta k(z,t,P) \equiv [1-\eta(z,t)]\cdot\Delta k_\mathrm{N}(P) + \eta(z,t)\cdot\Delta k_\mathrm{P}(P) + \Delta k_\mathrm{D}(z,t) + \Delta k_\mathrm{G}(z) \tag{3}$$

where η(z,t) is the time-and spatially-dependent fraction of ionized atoms, and P is the gas pressure. The first term above describes the mismatch arising from the dispersion of the refractive index n of the charge-*neutral* atomic gas,

$$\Delta k_\mathrm{N}(P) = \frac{q\omega_0}{c_0}\cdot[n(q\omega_0,P) - n(\omega_0,P)]. \tag{4}$$

In turn, the second term represents the mismatch due to the *plasma* dispersion of the ionized gas

$$\Delta k_{\mathrm{P}}(P) \approx \frac{q\omega_0}{c_0} \cdot \frac{\rho(P)\, e^2}{2\varepsilon_0 m_{\mathrm{e}} \omega_0^2} \left(1 - \frac{1}{q^2}\right), \tag{5}$$

with $\rho(P)$ being the total gas density, e the electron charge, and m_e the free electron mass. The approximation is valid as long as the plasma frequency is well below the driving field frequency, which is well fulfilled for our pressures. Equation (5) represents 100% ionization and is accordingly scaled by the ionized fraction $\eta(z,t)$ in Eq. (3). This plasma wavevector mismatch is dominated by the fundamental wave since the underlying Drude polarizability scales with $\approx 1/\omega^2$, resulting in a significant reduction of the term in UV-driven HHG.

The third term derives from the atomic *dipole* phase $\phi_D = -\alpha_q \cdot I(z,t)$, accumulated by the ionized electron wavepacket as it gets accelerated and returned to the parent ion in the strong laser field. The corresponding wavevector mismatch is:

$$\Delta k_{\mathrm{D}}(z,t) = -\alpha_q \frac{2z}{z_{\mathrm{R}}^2} \frac{1}{1 + \left(z/z_{\mathrm{R}}\right)^2} I(z,t), \tag{6}$$

where $\alpha_q = 1\times10^{-14}$ cm^2 W^{-1} for the short trajectory contribution relevant in this study.[5] Finally, the last contribution to the wavevector mismatch in Eq. (3) is the *Gouy* phase

$$\Delta k_{\mathrm{G}}(z) = \frac{q}{z_{\mathrm{R}}} \cdot \frac{1}{1 + \left(z/z_{\mathrm{R}}\right)^2}, \tag{7}$$

due to the difference in geometric phase shifts in the Gaussian beam focus of the fundamental and harmonic laser fields.

Ionization level. The time-dependent ionization level $\eta(z,t)$ at each location was calculated within the framework of the Yudin-Ivanov (YI) model.[6] The corresponding YI ionization rate is (in SI units):

$$w(z,t) = N(z,t) \exp\left\{ -\frac{e^2 E_{\mathrm{env}}(z,t)^2}{\hbar m_{\mathrm{e}} \omega_0^3} \Phi\big(\gamma(t), \theta(t)\big) \right\}, \tag{8}$$

with

$$N(z,t)=\frac{2^{2n^*}}{n^*\Gamma(n^*+l^*+1)\Gamma(n^*-l^*)}\times\frac{(2l+1)(l+|m|)!}{2^{|m|}|m|!(l-|m|)!}\times\sqrt{\frac{3\kappa}{\gamma(I)^3}}C_{\mathrm{PPT}}(\gamma(I),m)\frac{eI_{\mathrm{p}}}{\hbar}\left[\frac{\sqrt{32m_{\mathrm{e}}(eI_{\mathrm{p}})^3}}{\hbar e|E_{\mathrm{env}}(z,t)|}\right]^{2n^*-|m|-1}$$

(9)

where $E_{\mathrm{env}}(z,t)$ is the envelope part of the electric field and γ is the Keldysh parameter, governed by the ratio of the atomic ionization potential I_{p} and twice the ponderomotive energy U_{p} of the driving field, i.e.

$$\gamma^2(I)=\frac{I_{\mathrm{p}}}{2U_{\mathrm{p}}}=I_{\mathrm{p}}\Big/2\left(\frac{eE_{\mathrm{env}}^2(z,t)}{4m_{\mathrm{e}}\omega_0^2}\right)=c_0\varepsilon_0 m_{\mathrm{e}}\frac{I_{\mathrm{p}}\omega_0^2}{e\,I(z,t)}, \tag{10}$$

and moreover

$$\kappa=\ln\left(\gamma+\sqrt{\gamma^2+1}\right)-\frac{\gamma}{\sqrt{\gamma^2+1}}. \tag{11}$$

The exponent factor in Eq. (8) is per the YI model[6] given as

$$\Phi(\gamma,\theta)=\left(\gamma^2+\sin^2\theta+1/2\right)\ln c-\frac{3\sqrt{b-a}}{2\sqrt{2}}\sin|\theta|-\frac{\sqrt{b+a}}{2\sqrt{2}}\gamma, \tag{12}$$

with

$$a\equiv 1+\gamma^2-\sin^2\theta,\quad b\equiv\sqrt{a^2+4\gamma^2\sin^2\theta},\ c\equiv\sqrt{\left(\sqrt{\frac{b+a}{2}}+\gamma\right)^2+\left(\sqrt{\frac{b-a}{2}}+\sin|\theta|\right)^2}. \tag{13}$$

Note that in these expressions the phase θ of the driving field is defined as being wrapped within a π interval, such that $-\pi/2 < \theta(t) \leq \pi/2$. Like the cycle-averaged Perelomov-Popov-Terent'ev (PPT) theory,[7] the YI model encompasses arbitrary Keldysh parameters γ, starting from quasi-static tunneling ionization ($\gamma \ll 1$) described by the model of Ammosov, Delone and Krainov (ADK)[8] up to the limit of multi-photon ionization ($\gamma \gg 1$). The YI-model ionization rate is normalized such that the cycle-averaged rate equals that of the PPT model, and thus includes the PPT Coulomb correction factor

$$C_{\mathrm{PPT}}(\gamma,m)=\left(1+\gamma^2\right)^{\frac{m}{2}+\frac{3}{4}}A_m(\gamma), \tag{14}$$

with

$$A_m(\gamma) = \frac{4}{\sqrt{3\pi}} \frac{1}{|m|!} \frac{\gamma^2}{1+\gamma^2} \sum_{n \geq \nu_{\mathrm{TH}}(\gamma)}^{\nu_{\mathrm{MAX}}(\gamma)} e^{-\alpha(\gamma)\times\{n-\nu_{\mathrm{TH}}(\gamma)\}} \, w_m\left(\sqrt{\beta(n-\nu_{\mathrm{TH}}(\gamma))}\right). \tag{15}$$

In the above,

$$\alpha \equiv 2\left(\operatorname{asin}\gamma - \frac{\gamma}{\sqrt{1+\gamma^2}} \right), \tag{16}$$

$$\beta \equiv \frac{2\gamma}{\sqrt{1+\gamma^2}}, \text{ and} \tag{17}$$

$$w_m(x) \equiv \frac{x^{2|m|+1}}{2} \int_0^1 \frac{\exp(-x^2\tau)\tau^{|m|}}{\sqrt{1-\tau}} \, d\tau \, . \tag{18}$$

Furthermore, the limits of the sum are given by the quantum threshold and a reasonable convergence limit[4,7]

$$\nu_{\mathrm{TH}} = \operatorname{ceil}\left\{ \frac{eI_{\mathrm{p}}}{\hbar\omega_0} \times \left(1 + \frac{1}{2\gamma^2}\right) \right\} \quad \text{and} \quad \nu_{\mathrm{MAX}} = \nu_{\mathrm{TH}} + \operatorname{ceil}\left\{ \frac{10}{\alpha(\gamma)} \right\}. \tag{19}$$

To calculate the total ionization rate for each spatial position *z*, we solve Eq. (8) and related expressions, summing up the rates for the relevant highest atomic levels with $l = 1$, $m = 0,\pm1$. For Krypton, the ionization potential is $I_{\mathrm{p}} = 13.9996$ eV, from which correspondingly $n^* \equiv (2I_{\mathrm{p}})^{-1/2} = 0.98583$ and $l^* \equiv n^* - 1 = -0.01417$. The numerical calculation is sped up by taking into account that the integral in Eq. (18) can be expressed by Kummer’s confluent hypergeometric function ${}_1\mathrm{F}_1(a,b,z)$ as:

$$w_m(x) \equiv \frac{x^{2|m|+1}}{2} \sqrt{\pi} \, \frac{\Gamma(|m|+1)}{\Gamma\left(|m|+3/2\right)} \, {}_1F_1\left(|m|+1, |m|+\frac{3}{2}, -x^2 \right). \tag{20}$$

Moreover, $N(t)$ varies slowly and needs to be evaluated only once per cycle. For each spatial position, the resulting rate $w(t)$ with its sub-cycle dependence is integrated in time along a finely-discretized electric driving field $E(z,t)$ to obtain the ionization level η.

XUV Photon Flux and Enhancement Factor. We express the number of XUV photons generated in the q-th harmonic at pressure P as

$$N_q(P) \propto \beta_S(q, \omega_0) \times \xi_q(P), \tag{21}$$

Here, β_S is the single-atom efficiency (per harmonic) that scales with drive frequency approximately as ω_0^5 around the cutoff due to the wavepacket quantum diffusion, driving field energy, and harmonic spacing.[9] Moreover, ξ_q represents the enhancement factor arising from the spatio-temporal folding of XUV emission and phase-matching, obtained by integrating across the pulse duration and interaction volume:

$$\xi_q(P) \equiv \int_{-\infty}^{\infty} \left| \int_{z_{min}}^{z_{max}} \sqrt{w(z,t)}\ \rho(P) \cdot [1-\eta(z,t)] \cdot \exp\{-\alpha_F(z,t,P) + i\varphi(z,t,P\}\, dz \right|^2 dt, \tag{22}$$

following the models of C. Lai *et al.*[10] and E. Constant *et al*.[11] This takes into account the XUV emission via the ionization rate w,[10,12] neutral gas density ρ, and ground state depletion 1-η, as well as the XUV attenuation and phase mismatch. In the above, $\alpha_F(z,t,P) = \int_z^{z_{max}} (1-\eta(z,t))/2L_{abs}(q\omega_0)\, dz$ represents the field attenuation from each point z up to the exit of the medium. The absorption length $L_{abs}(\omega) = 1/\sigma(\omega)\rho(P)$ depends on the neutral gas density ρ and XUV absorption cross section σ. Furthermore, $\varphi(z,t,P) = \int_z^{z_{max}} \Delta k(z,t,P) dz$ is the phase difference accumulated from the emission at z to the medium exit. For the optical properties of Kr gas, the absorption cross section is $\sigma = 34$ Mb at 22.3 eV,[13] and the refractive index dispersion is modeled via the Sellmeier equation of Ref. 14.

Supplementary References

1. Shiles, E., Sasaki, T., Inokuti, M. & Smith, D. Y. Self-consistency and sum-rule tests in the Kramers-Kronig analysis of optical data: applications to aluminum. *Phys. Rev. B* **22,** 1612–1628 (1980).

2. French, R. H., Müllejans, H. & Jones, D. J. Optical properties of aluminum oxide : determined from vacuum ultraviolet and electron energy-loss spectroscopies. *J. Am. Ceram. Soc.* **81,** 2549–2557 (1998).

3. Born, M. & Wolf, E. *Principles of Optics*. Cambridge University Press, Cambridge (1999).

4. Chang, Z. *Fundamentals of Attosecond Optics*. CRC Press, Boca Raton (2011).

5. Varjú, K. *et al.* Frequency chirp of harmonic and attosecond pulses. *J. Mod. Opt.* **52,** 379–394 (2006).

6. Yudin, G. & Ivanov, M. Nonadiabatic tunnel ionization: looking inside a laser cycle. *Phys. Rev. A* **64,** 013409 (2001).

7. Perelomov, A. M., Popov, V. S. & Terent'ev, M. V. Ionization of atoms in an alternating electrical field. *Sov. Phys. JETP* **23,** 924–934 (1966).

8. Ammosov, M. V., Delone, N. B. & Krainov, V. P. Tunnel ionization of complex atoms and of atomic ions in an alternating electromagnetic field. *Sov. Phys. JETP* **64,** 1191–1194 (1986).

9. Falcão-Filho, E. L., Gkortsas, V. M., Gordon, A. & Kärtner, F. X. Analytic scaling analysis of high harmonic generation conversion efficiency. *Opt. Express* **17,** 11217–29 (2009).

10. Lai, C.-J. & Kärtner, F. X. The influence of plasma defocusing in high harmonic generation. *Opt. Express* **19,** 22377–22387 (2011).

11. Constant, E. *et al.* Optimizing high harmonic generation in absorbing gases: Model and experiment. *Phys. Rev. Lett.* **82,** 1668–1671 (1999).

12. Gordon, A. & Kärtner, F. X. Quantitative modeling of single atom high harmonic generation. *Phys. Rev. Lett.* **95,** 223901 (2005).

13. Kennedy, D. J. & Manson, S. T. Photoionization of the noble gases: cross sections and angular distributions. *Phys. Rev. A* **5,** 227–247 (1972).

14. Bideau-Mehu, A., Guern, Y., Abjean, R. & Johannin-Gille, A. Measurement of refractive indices of neon, argon, krypton, and xenon in the 253.7-140.4 nm wavelength range. Dispersion relations and estimated oscillator strengths of the resonance lines. *J. Quant. Spectrosc. Radiat. Transf.* **25,** 395–402 (1981).
